# Cost-effectiveness analysis of parenteral antimicrobials for acute melioidosis in Thailand

**DOI:** 10.1093/trstmh/trv002

**Published:** 2015-05-13

**Authors:** Viriya Hantrakun, Wirongrong Chierakul, Ploenchan Chetchotisakd, Siriluck Anunnatsiri, Bart J. Currie, Sharon J. Peacock, Nicholas P. J. Day, Phaik Cheah, Direk Limmathurotsakul, Yoel Lubell

**Affiliations:** a Mahidol-Oxford Tropical Medicine Research Unit (MORU), Faculty of Tropical Medicine, Mahidol University, Bangkok 10400, Thailand; b Department of Clinical Tropical Medicine, Faculty of Tropical Medicine, Mahidol University, Bangkok 10400, Thailand; c Melioidosis Research Centre, Department of Medicine, Faculty of Medicine, Khon Kaen University, Khon Kaen 4002, Thailand; d Royal Darwin Hospital and Menzies School of Health Research, Darwin, Northern Territory 0811, Australia; e Department of Medicine, University of Cambridge, Cambridge, CB2 1TN, UK; f Center for Tropical Medicine and Global Health, Nuffield Department of Clinical Medicine, University of Oxford, Oxford, OX3 7LE, UK; g Department of Tropical Hygiene, Faculty of Tropical Medicine, Mahidol University, Bangkok 10400, Thailand

**Keywords:** Antimicrobials, Ceftazidime, Cost-effective treatment, Meliodosis, Meropenem, Northeast Thailand

## Abstract

**Background:**

Melioidosis is a common community-acquired infectious disease in northeast Thailand associated with overall mortality of approximately 40% in hospitalized patients, and over 70% in severe cases. Ceftazidime is recommended for parenteral treatment in patients with suspected melioidosis. Meropenem is increasingly used but evidence to support this is lacking.

**Methods:**

A decision tree was used to estimate the cost-effectiveness of treating non-severe and severe suspected acute melioidosis cases with either ceftazidime or meropenem.

**Results:**

Empirical treatment with meropenem is likely to be cost-effective providing meropenem reduces mortality in severe cases by at least 9% and the proportion with subsequent culture-confirmed melioidosis is over 20%.

**Conclusions:**

In this context, treatment of severe cases with meropenem is likely to be cost-effective, while the evidence to support the use of meropenem in non-severe suspected melioidosis is not yet available.

## Introduction

Melioidosis is caused by the Gram-negative bacillus *Burkholderia pseudomallei.* The population mortality rate for melioidosis in northeast Thailand is comparable to TB and exceeds that of malaria and diarrheal illness combined.^1^ Mortality in hospitalized patients approximates 40% and in more severe cases it can exceed 70%.^1^ Its clinical features are variable and often indistinguishable from other infectious diseases. Diagnosis is based on bacterial culture and can take 2 to 7 days, during which suspected melioidosis cases are treated with an empirical antimicrobial.

Current recommended antimicrobial therapies for acute melioidosis are parenteral ceftazidime or a carabapenem for 10–14 days prior to oral radical therapy with trimethoprim-sulfamethoxazole for 12–20 weeks. A retrospective study in Australia suggested that meropenem might be associated with lower mortality compared to ceftazidime in patients with severe melioidosis.^2^ In Thailand, despite the absence of evidence from clinical trials, carbapenems are increasingly used in all patients with suspected melioidosis (Direk Limmathurotsakul, personal communication).

The potential overuse of carbapenems is concerning given their high cost in Thailand (approximately US$140/day as compared with US$5/day for ceftazidime) and increasing incidences of nosocomial infections with carbapenem-resistant organisms. In this paper, we explore conditions under which the use of meropenem for the treatment of suspected melioidosis could be cost-effective in northeast Thailand.

## Materials and methods

A decision tree was developed to evaluate the cost-effectiveness of treatments for acute melioidosis (see Supplementary Figure 1). The estimates and probable ranges for the percentage of culture-confirmed melioidosis in patients with suspected acute melioidosis, effectiveness of ceftazidime and other parameters were gathered from recent literature^3^ and expert opinion (see Supplementary Table 1). We evaluated three treatment plans, consisting of (Plan A) ceftazidime as an empirical treatment for all patients with suspected melioidosis, including both severe and non-severe cases; (Plan B) meropenem as empirical treatment for patients with suspected severe melioidosis and ceftazidime for patients with suspected non-severe melioidosis and (Plan C) meropenem as an empirical treatment for all patients with suspected melioidosis.

Costs included those for hospitalization and drugs and health benefits were based on expected years of life saved (LYS). The analysis identified configurations of parameter estimates for the percentage of culture confirmed cases in patients who were empirically treated, and for the relative reduction of case fatality rate (CFR) in which each plan would be cost-effective. The willingness to pay threshold per LYS was set at the Thai GDP per capita (approximately US$3000). The analysis was performed using TreeAge Pro 2013 (TreeAge Software Inc., Williamstown, MA, USA).

## Results

**Figure 1.**
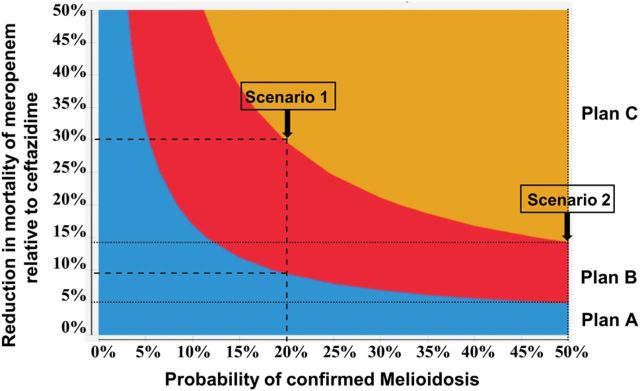
(Summary of the cost-effectiveness analysis showing scenarios in which ceftazidime or meropenem could be a cost-effective strategy for the treatment of patients with suspected melioidosis in Thailand). Plan A: Ceftazidime is used for empirical treatment of all suspected melioidosis cases; the point estimate cost of this strategy was US$706 (ranging between US$615 - US$767 in the sensitivity analysis). Plan B: Meropenem is used for empirical treatment of patients with suspected severe melioidosis, ceftazidime is used for suspected non-severe melioidosis; the strategy cost was US$1208 (range US$767- US$1871). Plan C: Meropenem is used for empirical treatment of all suspected melioidosis cases; the strategy cost was US$1799 (range US$1750 - US$1871). This figure is available in black and white in print and in color at Transactions online.

Figure 1 shows the configuration of parameter estimates for reduction in CFR and percentage of melioidosis-confirmed cases in which each strategy is cost-effective. In a scenario where 20% of empirically treated patients are culture-confirmed melioidosis, meropenem would be cost-effective as an empirical treatment for patients with suspected severe melioidosis (Plan B) and for all suspected melioidosis patients (Plan C) if meropenem reduced mortality by at least 9% (absolute reduction from 70% to at least 64%) and at least 30% (absolute reduction from 40% to at least 28%), respectively (Figure 1, Scenario 1). This proportion of culture-confirmation is a reasonable estimate for routine practice in northeast Thailand.

In a scenario where over 50% of empirically treated patients are culture-confirmed melioidosis, (Plan B) would be cost-effective with a reduction in CFR of 5% and (Plan C) with a reduction of at least 14%, respectively (Figure 1, Scenario 2). Such a high proportion of culture-confirmation has been reported in the context of clinical trials, however, this is unlikely to be representative of routine care.

## Discussion

Whether meropenem can reduce mortality in acute melioidosis remains unclear. The only published trial comparing a carbapenem with ceftazidime in severe cases found a modest and non-significant reduction in mortality.^4^ The outcomes of a clinical trial comparing ceftazidime and meropenem in severe melioidosis are not yet available (NCT00579956).

In addition to the scanty evidence are a number of other limitations. First, we assume general sepsis guidelines would be effective in classification of severity. If this is wrong, some non-severe cases could receive the more costly treatment unnecessarily, while more severe cases would be denied the potential advantage offered by meropenem. Second, we assume both treatments are equally effective in empirically treated non-melioidosis cases. Third, due to methodological challenges we did not include the societal cost of carbapenem-resistant organisms following increased use of meropenem.

Costs and other key parameter estimates are specific to the Thai context, therefore our recommendations may not be generalizable to other settings. However, the model could be adopted and re-analyzed with other sites' specific parameters to identify the cost-effective treatment strategies for acute melioidosis.

### Conclusions

Notwithstanding the limited evidence, we conclude that use of meropenem in patients with suspected severe melioidosis is likely to be cost-effective in northeast Thailand, assuming even a modest reduction in mortality. This strategy also resembles the standard guidelines of melioidosis treatment in Australia.^5^ Empirical meropenem treatment for all acute cases, however, is less likely to be cost-effective unless the reduction in mortality is much higher than indicated by a previous clinical trial.^4^ This analysis should be repeated once better evidence become available.

## Supplementary data

Supplementary data are available at Transactions Online (http://trstmh.oxfordjournals.org).

Supplementary Data
