# Supplementary material for: Cost-effectiveness analysis of parenteral antimicrobials for acute melioidosis in Thailand
Source: Trans R Soc Trop Med Hyg. 2015 May 13;109(6):416–8. doi: 10.1093/trstmh/trv002 (PMC4553702; doi:10.1093/trstmh/trv002)
Supplement: Supplementary Data [file supp_109_6_416__index.html]

Cost-effectiveness analysis of parenteral antimicrobials for acute melioidosis in Thailand — Supplementary Data 

# Cost-effectiveness analysis of parenteral antimicrobials for acute melioidosis in Thailand

## Supplementary Data

Supplementary Data

- Supplementary Data - Docx file
