# Supplementary material for: Cost-effectiveness analysis of parenteral antimicrobials for acute melioidosis in Thailand
Source: Trans R Soc Trop Med Hyg. 2015 May 13;109(6):416–8. doi: 10.1093/trstmh/trv002 (PMC4553702; doi:10.1093/trstmh/trv002)
Supplement: Supplementary Data [file supp_trv002_trv002supp.docx]

**Supplementary Figure 1. The decision model of the possible treatment plans for patients suspected of acute melioidosis in Thailand.**

| **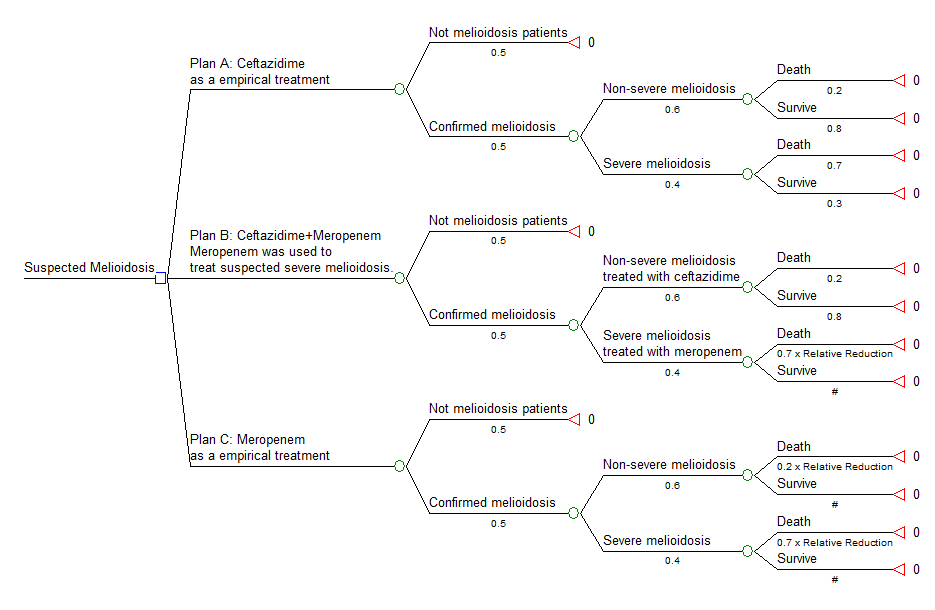** |
| --- |

The decision tree models the course of treatment for all suspected melioidosis cases and the outcomes of three treatment strategies. Plan A uses ceftazidime as an empirical treatment versus Plan B where meropenem is used as empirical treatment only for patients with suspected severe melioidosis and Plan C where meropenem is used as an empirical treatment for non-severe and severe suspected melioidosis.

**Supplementary Table 1. Summary of the input parameters used in the decision model**

| **Input parameters** | **Values**  **(range)** | **References** |
| --- | --- | --- |
| 1. Proportions and case fatality rate |  |  |
| - Proportion of culture-confirmed melioidosis in patients who were suspected of and treated as melioidosis | 0.2  (0.05-0.5) | Expert opinions |
| - Proportion of melioidosis cases that are severe | 0.4  (0.2-0.8) | Expert opinions |
| - Case fatality in non-severe melioidosis treated with ceftazidime | 0.2  (0.1-0.3) | Limmathurotsakul et al. (2010)[^1^](#_ENREF_1) and expert opinions |
| - Case fatality in severe melioidosis treated with ceftazidime | 0.7  (0.5-0.9) | Cheng et al. (2007)[^2^](#_ENREF_2) and expert opinions |
| 1. Costs |  |  |
| - Cost of hospitalization per day | US$57 | Riewpaiboon et al. (2011) |
| - Cost of ceftazidime per day | US$5 | Sappasithiprasong hospital (2011) |
| - Cost of meropenem per day | US$140 | Sappasithiprasong hospital (2011) |
| 1. Length of hospitalization |  |  |
| - For non-severe melioidosis | 4 | Model assumption |
| - For non-severe melioidosis cases who died | 6.5  (0-41 days) | Dataset of Chierakul et al. (2005)[^3^](#_ENREF_3) |
| - For non-severe melioidosis cases who survived | 14.5  (2-52 days) | Dataset of Chierakul et al. (2005)[^3^](#_ENREF_3) |
| - For severe melioidosis cases who died | 4.5 (0-17 days) | Dataset of Chierakul et al. (2005)[^3^](#_ENREF_3) |
| - For severe melioidosis cases who survived | 23  (7-56 days) | Dataset of Chierakul et al. (2005)[^3^](#_ENREF_3) |
| 1. Length of treatment with parenteral drugs |  |  |
| - For non-severe melioidosis | 4 | Model assumption |
| - For non-severe melioidosis cases who died | 3.5  (0-13) | Dataset of Chierakul et al. (2005)[^3^](#_ENREF_3) |
| - For non-severe melioidosis cases who survived | 8  (0-31) | Dataset of Chierakul et al. (2005)[^3^](#_ENREF_3) |
| - For severe melioidosis cases who died | 5.5  (1-18) | Dataset of Chierakul et al. (2005)[^3^](#_ENREF_3) |
| - For severe melioidosis cases who survived | 17  (8-29) | Dataset of Chierakul et al. (2005)[^3^](#_ENREF_3) |

**References**

1. Limmathurotsakul D, Wongratanacheewin S, Teerawattanasook N et al. Increasing incidence of human melioidosis in Northeast Thailand. Am J Trop Med Hyg 2010;82:1113-7.

2. Cheng AC, Limmathurotsakul D, Chierakul W et al. A randomized controlled trial of granulocyte colony-stimulating factor for the treatment of severe sepsis due to melioidosis in Thailand. Clin Infect Dis 2007;45:308-14.

3. Chierakul W, Anunnatsiri S, Short JM et al. Two randomized controlled trials of ceftazidime alone versus ceftazidime in combination with trimethoprim-sulfamethoxazole for the treatment of severe melioidosis. Clin Infect Dis 2005;41:1105-13.
